# Music to Their Ears: Reducing Antipsychotic Use With a Personalized Music Intervention for Rural Veterans

**DOI:** 10.7759/cureus.73232

**Published:** 2024-11-07

**Authors:** Tara Downs, Jaime Wilson, Sherry Brewer, Karla Miller, Melissa Swee, Virginia Taylor, Issis Betts-Jimenez, Janna Imel, Cassie Graham

**Affiliations:** 1 Pharmacy, Lexington Veterans Affairs Health Care System, Lexington, USA; 2 Veterans Health Administration Rural Scholars Fellowship, Office of Rural Health Veteran Rural Health Resource Center, Iowa City, USA; 3 Nursing, College of Nursing, University of Iowa, Iowa City, USA; 4 Integrative Medicine, AndHealth, Columbus, USA; 5 Rheumatology, Veterans Affairs Salt Lake City Health Care System, Salt Lake City, USA; 6 School of Medicine, University of Utah, Salt Lake City, USA; 7 Nephrology, University of Iowa Hospitals and Clinics, Iowa City, USA; 8 Nephrology, Iowa City Veterans Affairs Medical Center, Iowa City, USA; 9 Geriatrics, Lexington Veterans Affairs Health Care System, Lexington, USA; 10 Psychology, Lexington Veterans Affairs Health Care System, Lexington, USA

**Keywords:** antipsychotic, dementia, deprescribing, music intervention, rural areas

## Abstract

Background

This project investigates a music intervention to deprescribe antipsychotics in rural Veterans with dementia.

Methods

The Veterans Health Administration Home-Based Primary Care Program is care provided in the home by an interdisciplinary team with the goals of decreasing hospitalizations and falls, providing education to patients and caregivers, and improving quality of life. Eighteen Home Based Primary Care Veterans with dementia and active antipsychotic prescriptions were identified with the goal to deprescribe antipsychotics in 50% of them using a music intervention. Individualized playlists and assessments for Veteran quality of life and caregiver burden were evaluated. Phone visits tracked music utilization and captured the voice of the customer.

Results

Antipsychotic dose reduction occurred in five of eight Veterans, totaling eight dose reductions and one discontinuation. Veteran quality of life improved; however, caregiver burden increased initially. The caregiver burden did improve when an outlier was removed. The voice of the customer favored music intervention.

Conclusions

A personalized music intervention is a feasible approach for reducing antipsychotic use in rural Veterans, improving quality of life, and potentially reducing caregiver burden.

## Introduction

Antipsychotic (AP) use for behavioral and psychological symptoms in dementia (BPSD) has been linked to an increased risk of death [1] and has recently been associated with increased risks of stroke, venous thromboembolism, myocardial infarction, heart failure, fracture, pneumonia, and acute kidney injury [2]. A closer examination of the use and potential safe alternatives in the home is warranted [1,2].

Alzheimer’s dementia (AD) remains the fifth-leading cause of death among Americans aged 65 and older [3]. There are more than 6.7 million Americans living with AD and 13.8 million people aged 65 and older are projected to have AD by 2060. AD is one cause of dementia and is the most common cause, accounting for approximately 60-80% of cases [3]. For the purpose of this paper, the term dementia will be utilized moving forward as all-encompassing for a particular group of symptoms with different causes [3]. Over 11 million Americans provide unpaid care for individuals with dementia. These caregivers provided more than 18 billion hours of care valued at nearly $339.5 billion in 2022 [3], resulting in significant caregiver burden and stress [3]. This burden increases when behavioral changes occur in the person with dementia [3]. Efforts are needed to improve patient access to nonpharmacological interventions and to provide education to caregivers on the use of these interventions to assist in the management of BPSD [1].

Antipsychotics (APs) are often used to treat agitation and aggressive behavior when there is potential for harm to the dementia patient or others. APs may also be prescribed in dementia patients for their perceived potential to ease daily care, improve sleep, or relieve caregiver burden [4]. Overuse of AP is also a concern and use is often continued chronically, despite a lack of compelling indications [4]. Literature supports a trial of deprescribing AP after ~ 3 to 4 months of use if the patient is stable, symptoms are unchanged, or if the indication for use is insomnia [4].

Non-pharmacologic interventions, specifically music therapy, have been shown to decrease agitation, aggression, pain, and the need for AP medications in a nursing home or inpatient setting [5-11]. Such nonpharmacologic alternatives are less widely used due to limited access, the need for a high number of resources, and additional healthcare staff/caregiver training. When considering music therapy, it is important to highlight descriptive terminology for music-based interventions [12]. Music therapy is an established health profession in which music is used within a therapeutic relationship and includes the triad of music, clients, and qualified credentialed music therapists. By contrast, music medicine is defined as having patients listen to prerecorded or live music, which is often managed by a medical professional other than a music therapist, such that the music plays the role of a medicine (referred to as music intervention in this project) [12]. Evidence is lacking to determine if a music intervention is beneficial for rural homebound Veterans on AP with BPSD.

The Veterans Health Administration (VHA) Home-Based Primary Care (HBPC) Program is care provided in the home by an interdisciplinary team with goals of decreasing hospitalizations and falls, providing education to patients and caregivers, and improving quality of life. HBPC is comprised of an aging Veteran population where the incidence of dementia is on the rise. The Lexington Veteran Affairs Health Care System (LVAHCS) HBPC program has a highly rural catchment area of 43 counties, includes nine Patient Aligned Care Teams (PACT), and the census at the time of the project’s inception was approximately 650 Veterans. It is estimated that around 30% of these Veterans have a diagnosis of dementia [12,13]. 

LVAHCS HBPC is recognized by the Institute for Healthcare Improvement (IHI) as an Age-Friendly Health System - Committed to Care Excellence, which aligns with VHA’s vision to become the largest integrated health system in the United States with this recognition. Age-Friendly Health Systems utilize “The 4Ms - What Matters, Medication, Mentation, Mobility” framework, implemented together, to guide the care of older adults wherever and whenever they encounter the health system. What matters to many of these rural Veterans is to remain in their home for as long as possible [13].

The LVAHCS HBPC team identified an area of concern with the extended use of APs for BPSD in rural Veterans within the service. Although many barriers were identified to deprescribing APs, the team decided to focus on improving nonpharmacologic options for the treatment of BPSD available in the home setting. After a review of the literature [5-10] and with knowledge of success in our own Community Living Center, a VA Nursing Home, the team chose to implement a music intervention via the Music and Memory Program© [14]. The transformation goal of this project was to improve Veteran care/safety, decrease caregiver burden, enhance quality of life, and implement a music intervention in a rural healthcare setting.

Forty veterans enrolled in HBPC had a diagnosis of dementia and an active prescription for an AP. Approximately 50% of those veterans did not carry a compelling indication for AP use. Our specific, measurable, attainable, relevant, and time-oriented (SMART) aim was set to decrease the dose of AP in 50% of those Veterans using a three-month music intervention.

This project received a determination of not research by the LVAHCS Institutional Review Board.

## Materials and methods

Model for improvement

Participants

Veterans were identified for inclusion using VIONE (Vital, Important, Optional, Not indicated, and Every medication has an indication) Potentially Inappropriate Medications (PIM) Deprescribing Dashboard, a practical methodology designed to facilitate a patient-centered approach to optimizing medication therapy and reducing potentially inappropriate medications [15], then cross-referenced by a local report created by the Pharmacy Automated Data Processing Application Coordinator (ADPAC). Veterans identified in both reports had their charts reviewed by the Clinical Pharmacy Practitioner (CPP). Veterans were then included for consideration of music intervention if there was an active AP prescription with an indication of BPSD/insomnia for >90 days and with no additional compelling indications for AP use [4]. Prior to Veteran enrollment, a discussion of each Veteran occurred between the CPP and the provider for music intervention participation approval. Approval resulted in the notification of the Veterans PACT team psychologist. The psychologist obtained Veteran/caregiver consent to participate in the music intervention and obtained an individualized music list, QUALID (Quality of Life in Late Stage Dementia) score [16], and ZBI (Zarit Burden Interview) score [17] via face-to-face and VA video connect visits. Veteran enrollment from the pilot PACT began in November of 2022. Enrollment expanded to all HBPC PACTs in January 2023.

Funding and Leadership Buy-In 

The implementation process began in August 2022. The team obtained $7260.00 in funding for the Music & Memory© [14] Program (M&M) plus equipment from the local facility, Whole Health Funds. Funding for the purchase of iTunes (Apple, Inc., Cupertino, USA) was provided by the local Voluntary Services to the amount of $150.00. In September, the team received a non-research determination letter for this project. The team then created a Standard Operating Procedure for this service, approved by the local Office of Public Affairs.

Technology

Equipment selection was a very important step in implementation. Given rural barriers to broadband network connectivity in homes and to ease the use of intervention for caregivers, our team utilized MP3 players complete with individualized playlists as the music intervention. To gain the ability to download music to non-VA network devices, we worked closely with Information and Technology to prevent any security issues. This included obtaining approval for the procurement and use of a non-VA network computer to house a music library. This was a very important step in our implementation process as literature supports the most successful outcomes with personalized music [14]. To support music anti-piracy laws, MP3 players were to be returned to the facility once the Veteran was discharged from our HBPC program. Based on knowledge of limited, if any, medical equipment returns to the local facility, the team obtained approval from Infection Control for the return of equipment procedures for Veteran safety.

Training

Interprofessional staff training (which included members of the HBPC Dementia Subcommittee and psychologists) for the Music & Memory© [14] Program took place in October 2022. This 1.5-hour training was provided virtually. The training provided best practices for discovering music preferences at home. The training highlighted the value of exposing the Veteran to music and provided insight on evaluating positive verbal and nonverbal cues to note a positive response.

Outcome Measure

The primary aim of this project was to deprescribe antipsychotics in 50% of eligible Veterans during a three-month music intervention. Deprescribing was defined as antipsychotic dose reduction or discontinuation at any time during the music intervention.

Process Measures

The following were assessed both pre- and post-intervention: self-report of music use, Veteran quality of life (QOL) using the Quality of Life in Late-Stage Dementia (QUALID) Score [16], and caregiver burden using the Zarit Caregiver Burden Interview Score (ZBI) four-item score [17].

Balancing Measures

Antipsychotic re-prescribing, defined as an increase in AP dose or restart if AP had been discontinued during the intervention period, as well as the addition of any additional medication potentially used for BPSD, defined as antidepressants, mood stabilizers, hypnotics, and anxiolytics, were documented.

Product Dissemination

The individualized music recommendations, with a focus on favorites from the Veteran's formative years (ages 10 to 25), were provided to the centrally located CPP, who used those recommendations to curate a playlist containing 75 to 100 songs, downloaded those songs to an MP3 player, and disseminated the MP3 player with additional music kit equipment to our local facility warehouse. The M&M© At-home kit contained an MP3 player, headphones, Bluetooth speaker, spiral-bound M&M at-home guide, equipment instructions, flash drive with videos, and helpful resources (Appendices). A pre-established process was utilized to deliver the music kit to the local VA Outpatient Clinic nearest to the psychologist or the Veteran. The pre-established process included the use of a delivery truck that transports supplies to each local VA Outpatient Clinic on a consistent weekly basis. The psychologist picked up the M&M kit (Appendices) at the nearest VA Outpatient Clinic and then delivered it to the Veteran and demonstrated its use.

Follow-Up

Telephone visits were completed each month of the intervention by the CPP with the caregiver to assess the utilization of music, and Voice of the Customer (VOC) data were documented. Recommendations were then made in the electronic medical record to the provider to deprescribe the AP, if applicable. Once the three-month intervention was complete, the psychologist obtained the post-QUALID and ZBI scores. Data collected were documented in the Computerized Patient Record System (CPRS) and stored on a secured-drive, password-protected Microsoft Excel spreadsheet (Microsoft Corporation, Redmond, USA). Caregivers were called every month to self-report music use by answering three standardized questions about daily use, weekly use, and session time.

## Results

Outcome Measure

**Figure 1:**
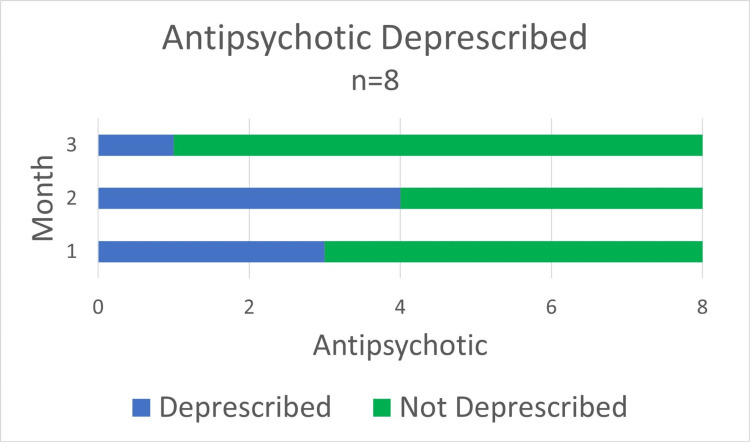
Deprescribed Antipsychotics Visual representation of antipsychotic deprescribing with the use of a music intervention during each month of the intervention.

Antipsychotics were deprescribed in five of eight Veterans for a total of eight dose reductions and one discontinuation (Figure 1). Only eight of the 18 veterans enrolled completed the three-month music intervention.

Process measures

Music utilization averaged 1752 ± 3404.7 minutes per week, with a range from 20 minutes per week to unlimited with breaks to perform activities of daily living. Unlimited music use is represented numerically as 10080 minutes per week. The ZBI mean pre- and post-intervention score increased from 7.13 ± 1.2 to 7.25 ± 2.5. The possible score range for ZBI is 0 to 16 with a score of 8 or higher indicating high burden [17]. Note that lower scores represent a lower burden. There was an outlier identified with a significant increase in ZBI score pre/post-intervention (Table 1).

**Table 1:** Zarit Caregiver Burden Interview (ZBI) Score *Assessment of caregiver burden with the use of Zarit Caregiver Burden Interview Score (ZBI) before and after the three-month music intervention. The possible score range for ZBI is 0 to 16 with a score of 8 or higher indicating high burden. Average score with standard deviation reported. A lower score corresponds with a lower burden. **data with removal of outlier

	Pre-Intervention	Post-Intervention
ZBI 4 item screen, M (SD)	7.13 (1.2)	7.25 (2.5)
ZBI 4 item screen, M (SD)**	7.43 (1)	6.86 (2.4)

**Figure 2:**
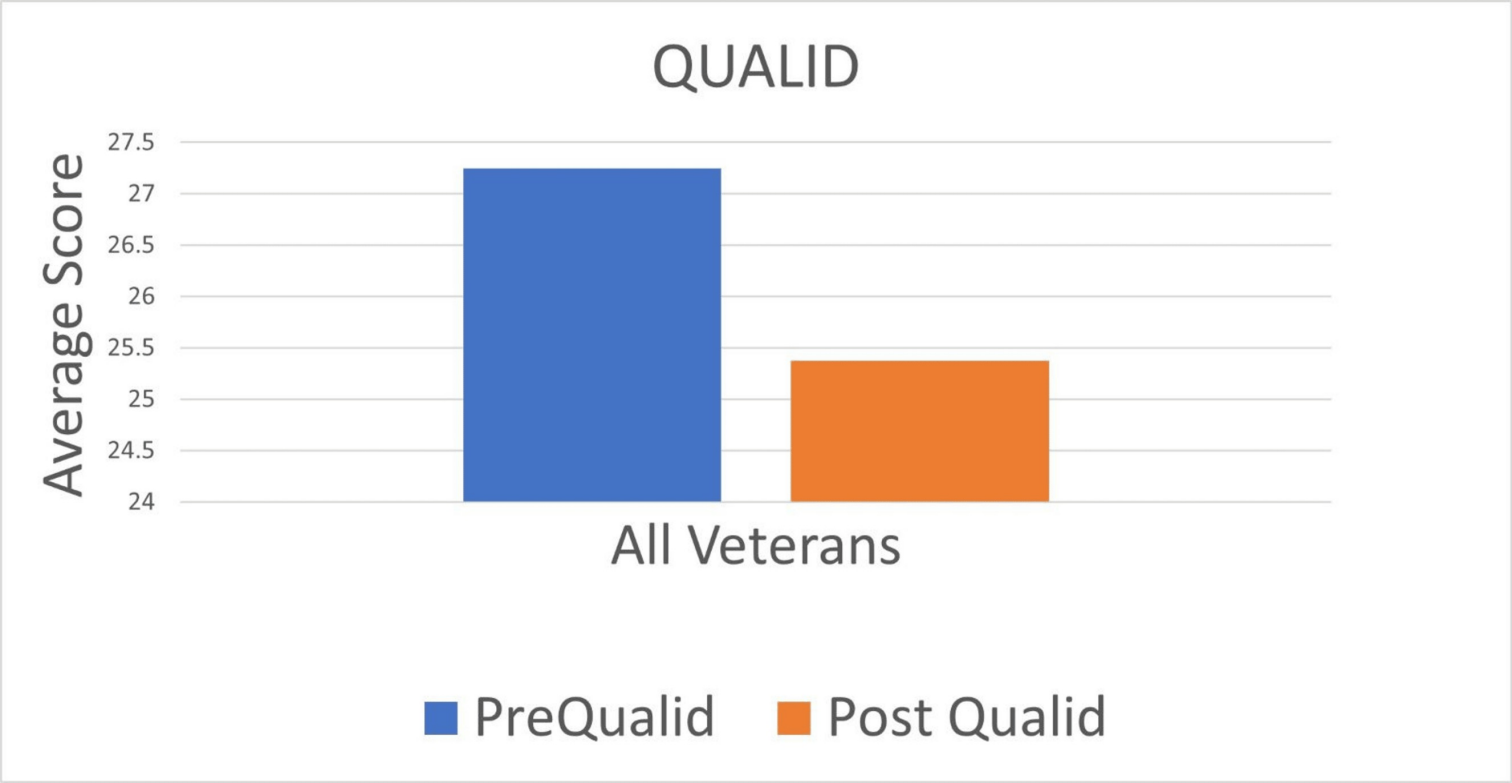
Quality of Life in Late-Stage Dementia Score Graphic demonstration of the Quality of Life in Late-Stage Dementia (QUALID) score before and after the three-month music intervention in eight Veterans that completed the three-month music intervention. Average QUALID score noted on the X-axis. QUALID score ranges from 11 to 55, with lower scores representing a better quality of life.

The QUALID score decreased in four Veterans, increased in three Veterans, and was unchanged in one Veteran, demonstrating an improvement in the QOL of four veterans from 27.25 ± 4.2 to 25.4 ± 4.7 (Figure 2).

The possible score range for QUALID is 11 to 55, with 11 representing the highest quality of life [16]. Note that lower scores represent a better QOL.

Balancing measures

One Veteran had their AP re-prescribed. One Veteran received an additional anxiolytic (hydroxyzine) added during the intervention period. Informal VOC data was collected by the CPP during monthly phone calls to the caregivers. Many positive responses were noted (Table 2).

**Table 2:** Voice of the Customer Voice of the customer (VOC) data documented during telephone call intervention with caregiver and healthcare team member with notation of agreement to deprescribe antipsychotic (AP).

VOC #	Description	Caregiver Agreeance to AP Deprescribing
1	Music has a calming effect on the Veteran, making them feel "mellowed out."	Not specified
2	The Veteran displays less irritability when exposed to music.	Agreeable
3	The caregiver reports a significant reduction in the Veteran's agitation, particularly in the evenings, since incorporating music into their routine.	Agreeable
4	Music engagement led to increased cooperation from the Veteran during essential care activities like bathing and changing clothes, relieving the caregiver and nurse from challenges.	Agreeable
5	The Veteran did not express pain while listening to music, a departure from their usual pattern of constant pain verbalization while awake.	Not specified
6	Music is recognized as highly beneficial for the Veteran's well-being.	Not specified
7	The caregiver expresses gratitude for the Music and Memory program, highlighting its positive impact on the Veteran's engagement and enjoyment. They appreciate the program's variety and accessibility, hoping it can continue for the Veteran's benefit.	Agreeable

Note that of the positive VOC reports, four of seven caregivers were agreeable to AP deprescribing, defined as AP dose reduction, with one AP discontinued.

Product Dissemination

This process on average took approximately 30 days from the time of enrollment to delivery.

## Discussion

This music intervention in rural Veterans with dementia prescribed AP for BPSD adds practical knowledge to the literature. It is important to again review descriptive terminology for music-based interventions [12]. Music therapy is an established health profession in which music is used within a therapeutic relationship and includes the triad of music, clients, and qualified credentialed music therapists. By contrast, music medicine is defined as having patients listen to prerecorded or live music, which is often managed by a medical professional other than a music therapist, such that the music plays the role of a medicine (referred to as music intervention in this project) [12]. There is a paucity of literature on music intervention in the home. Two home-based music interventions for people with dementia and their caregivers were identified [18,19] and one included movement in treatment combination [19]. Veteran enrollment in the intervention provided unanticipated challenges. Only eight of the 18 Veterans enrolled completed the three-month music intervention as three Veterans declined, three Veterans expired, one caregiver gave the equipment away, one caregiver was unable to complete the QUALID/ZBI scores, and two Veterans experienced a transition to a higher level of care. 

The study did not meet our primary aim, however, a trend in AP deprescribing with the use of a music intervention was noted. Our sample was not large enough to apply statistical analysis. Our findings in the deprescribing of antipsychotics with the use of a music intervention are consistent with Bakerjian et al. and Parajuli et al., however, the settings are not comparable [5,6]. Bakerjian et al. reported that the odds of AP use declined by 11% with statistical significance in a residential setting [5]. Additionally, Parajuli et al. noted a downward trend in AP use in a rural residential setting [6]. These changes were not statistically significant, with small sample sizes noted as possible contributors [6]. Our project identified caregiver resistance to AP deprescribing as a major barrier, as this was the most frequent reason reported in caregiver interviews during music utilization assessments. Potentially, there are many reasons why a caregiver might be resistant. The team hypothesizes that the fear of the return of BPSD is a major contributor. This is perhaps less of a barrier in the institutional setting, where healthcare staff are likely equipped with more training and support to manage a return of BPSD. As Parajuli et al. highlight, the potential reason to support an association of a small sample size for the non-significant reduction in the use of psychotropic medications in their findings was that all the participating staff highlighted a less need for medications due to the reduction in BPSD of the residents [6].

Of the five Veterans who had their AP deprescribed, only one had AP re-prescribed during the intervention. This caregiver reported that the Veteran had increased pain occurring at similar times of AP reduction. This increase in pain was defined as an adverse drug withdrawal event (ADWE), and the AP was re-prescribed. This Veteran was later found to have a tibia fracture, and its treatment resulted in a Community Living Center (CLC) stay where the AP was successfully discontinued.

Successful AP deprescribing occurred in Veterans who utilized music > 350 minutes/week, with one outlier of success in a Veteran with an average of 160 minutes/week. These findings suggest that a personalized music intervention in the home may be associated with a decrease in AP use. The cessation of music listening by the Veteran classified as having an ADWE had a notable impact on the mean listening duration from month 2 to month 3. Similarly to The Roth Project [18], more frequent education to the caregiver in regards to when to use music may increase utilization rates. Our project did provide Bluetooth speakers to overcome potential barriers anticipated with headphones in Veterans with dementia, a recommendation noted in The Roth Project [18].

Notably, only one Veteran received an additional anxiolytic (hydroxyzine) and had their AP re-prescribed during the intervention period. This was thought to be associated with an acute event identified post-intervention.

Quality of life improvements were noted in QUALID scale scores for Veterans with the use of music. One Veteran had a significant score decrease of 14 points, which suggests improved QOL. This Veteran additionally had their AP successfully deprescribed. Of the three Veterans who experienced a decrease in QOL, i.e., a numerical increase in QUALID score, one Veteran experienced an acute event. These findings are consistent with other individuals with dementia who reside in an assisted living facility [9] that utilized a variation of the Music & Memory program. Additionally, The Roth Project also reported an increase in participants’ overall happiness, although a specific quality-of-life assessment was not utilized [18].

ZBI scores did not demonstrate a decrease in caregiver burden with this intervention. However, ZBI scores did improve in five of eight caregivers and remained unchanged in one. It is important to highlight the outlier with a large increase (+5) in score impacting the mean post-intervention scores. After removing the outlier, the mean of the ZBI scores pre- and post-intervention decreased from 7.4±1 to 6.9±2.4. One meta-analysis did note a study that demonstrated a possible increase in caregiver satisfaction; however, this was with the use of music therapy [20], whereas our study utilized a music intervention. Interestingly, The Roth Project [18] noted a positive caregiver response. Our VOC data enhance quantitative findings when considering the positive impact noted by caregivers. As our sample size was small, the team had knowledge of caregivers’ challenges, such as a decline in personal health, change in living situation, and/or new acute medical diagnoses of the Veteran during the intervention period. These caregivers were noted to have an increase in the ZBI score, corresponding with an increase in burden. 

The high potential for positive impact on care in this rural population is demonstrated in the VOC. Music utilization allowed the healthcare team members to perform a full physical exam on a Veteran who had previously been resistant. Music utilization also allowed the healthcare team to assist the caregiver in activities of daily living, such as changing soiled clothes and bathing. Additionally, another Veteran consistently verbalized pain while awake. It was noted that the verbalization of pain ceased while listening to music. Music's positive impact on pain, while not assessed in our study, has also been reported in the literature [20].

Limitations

The sample size was small and fluid and relied on caregiver self-report of music utilization. The team hypothesized that the enrollment time and morbidity/mortality of our HBPC population prevented the enrollment of the target number of Veterans. Surprisingly, some caregivers and Veterans declined participation. Our program also experienced high staff turnover during our intervention period, adding to the enrollment challenges. Additionally, the intervention period was short and there were many uncontrolled confounders. These limitations have been identified as gaps in this area of research [20].

Strengths

The real-world rural setting in the homes of Veterans is a major strength of this study. Additionally, the nonpharmacologic measure selected for use (music) was low cost and low risk when considering potential adverse outcomes, consistent with findings from The Roth Project [18].

Future directions

Future efforts should be aimed at equipping healthcare team members with the ability to empower caregivers, with available bandwidth and resources, to engage in the self-implementation of a music intervention. Future research should examine the barriers to caregiver resistance to deprescribing antipsychotics in this setting and the long-term impact of music intervention.

## Conclusions

This project demonstrates the feasibility of implementing low-cost, low-risk non-pharmacological music interventions to reduce AP use in rural Veterans with BPSD receiving home care. The VOC and quantitative data show a positive impact of personalized music interventions on Veterans’ QOL and also potentially on caregiver burden.

## Figures and Tables

**Figure 3:**
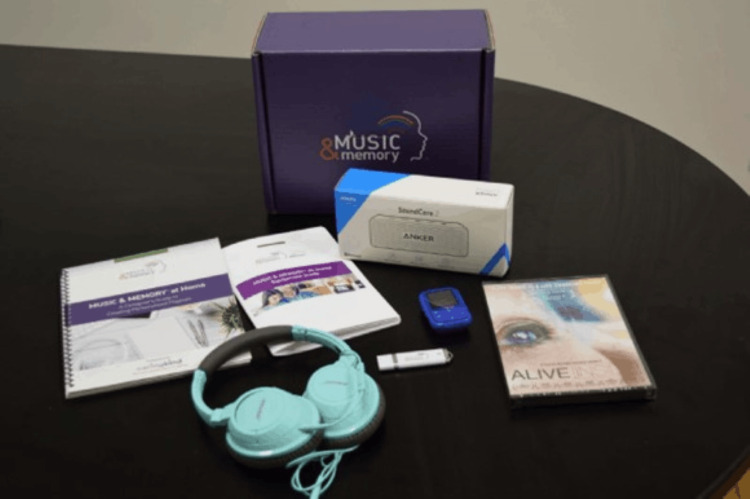
Music and Memory Music Kit https://musicandmemory.org/get-trained/home-training/
